# Automated Querying of Genome Databases

**DOI:** 10.1371/journal.pcbi.0030001

**Published:** 2007-01-26

**Authors:** Peter Schattner

**Affiliations:** Whitehead Institute, United States of America

## Introduction

The number of molecular biology databases continues to explode. Presently, few problems in genomic molecular biology can be addressed without analyzing data stored in them. However, these databases reside in many different locations and often use nonstandard data formats requiring specialized data parsers. As a result, integrating and comparing data from multiple biological databases is difficult and tedious. Genome databases offer solutions to this problem by integrating data from multiple databases in a uniform and standardized manner. However, principally because of the intrinsic complexity of genome data, exploiting the full power of these databases also has a considerable learning curve. This is particularly true if one wants to query multiple genomic regions in an automated manner, rather than simply analyze individual genes, one at a time. Here, using the University of California Santa Cruz (UCSC) Genome Database for illustration, I describe tools that have been developed for facilitating automated, genome-database–querying and present some applications for which they are well-suited.

The genome browsers at UCSC [1], Ensembl [2], and the National Center for Biotechnology Information (NCBI) [3], as well as the model organism databases (e.g., Wormbase [4], Flybase [5], Saccharomyces Genome Database (SGD) [6], and Mouse Genome Database (MGD) [7]), have become essential tools for the analysis of genomic, molecular biology data. By integrating data from multiple biological databases and visually displaying the results, these tools enable the exploration of relationships among genomic data in ways that were previously not possible. The power of this approach can be illustrated by a simple example. We can imagine a scenario in which we have found a polymorphism in a human disease gene and want to check various properties of this polymorphism. Is it in the Single Nucleotide Polymorphism Database (dbSNP), indicating that it has been previously identified? Does it overlap a known repeat sequence? Does it overlap a CpG island? Does it occur in any known expressed sequence tag (EST)? Is the more common variant at the polymorphism site conserved in other vertebrates? It is of course possible to answer these questions without a genome browser. However, this approach requires identifying, becoming familiar with, and using multiple different resources—dbSNP, NIH Genetic Sequence Databank (GenBank), the Expressed Sequence Tag Database (dbEST), etc. [3]—each with its own idiosyncrasies and learning curves. In contrast, using one of the genome browsers, all we need to do is to select the appropriate genome and genomic location, select the browser annotation tracks for SNPs, ESTs, repeats, CpG islands, and interspecies conservation, and view the results.

However, for all its power and convenience, interactive querying of genome databases does have limitations. For example, we can imagine a situation where we have identified *one hundred* polymorphisms rather than just a single one. Interactively verifying which ones are already in dbSNP, or are within CpG islands, or are represented by ESTs, or are at highly conserved sites would quickly become tedious, time-consuming, and error prone, even with an integrated genome browser. Similarly, if one has identified hundreds, or even thousands, of genome locations of interest from a microarray experiment, and wants to ask a set of biological questions about each of them, interactive querying is not feasible.

Moreover, without batch-querying, many important biological questions cannot practically be addressed at all. One might want to search for new genes, by looking for regions where ESTs overlap gene predictions and also are highly conserved among related species. Or one might want to study exon evolution, by searching for ALU repeat sequences found in coding exons [8]. Yet another example occurs in the identification of candidate target sites for adenosine deaminase enzymes that convert specific adenosines in RNA to inosine (ADARs). One effective method [9] for identifying ADAR sites is by searching for genomic locations that code for an A while a G has been observed at the corresponding location in an mRNA or EST (inosine is generally interpreted as guanosine by both reverse transcriptase and the ribosome). Such queries can be addressed in a straightforward manner by batch-querying the genome databases. In contrast, they are essentially impossible to answer via interactive genome browsing.

Consequently, both the UCSC and Ensembl Genome Browsers include tools for direct and automated “batch”-querying of their underlying databases. Currently the NCBI MapViewer Browser and the model organism databases do not offer integrated batch-querying tools, but NCBI is planning to introduce such tools for the MapViewer Database in the future (D. Church, personal communication). The aim of the present work is to introduce the reader to the available, batch-querying tools, to illustrate some of their features and capabilities, and to indicate the types of applications for which they are useful. The reader is assumed to be familiar with interactive use of at least one of the genome browsers. No programming experience is required for performing basic batch-querying procedures. However, for the more complex tasks described, experience with some computer language such as C, Perl, or Java, as well as familiarity with database-querying, i.e., Structured Query Language (SQL) [10], is necessary. For simplicity, much of the discussion will be focused on a single database, the UCSC Browser Database. However, comparable capabilities are available via Ensembl.

## Interactive Batch Database–Querying

**Figure 1:**
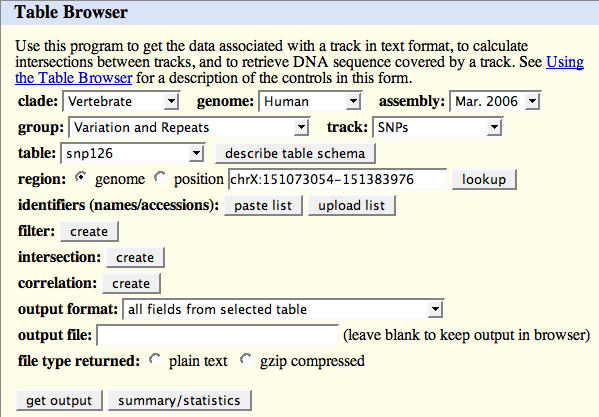
Table Browser Interface As shown in the screenshot, from the Table Browser Interface, one can select essentially any table in the UCSC Genome Database and obtain descriptions of the table's fields as well as download all or part of the table's records in a variety of formats.

Batch queries of the Ensembl and UCSC Genome Databases are possible using the conventional database SQL [10] or using the Application Programmer Interfaces (APIs), described below. Moreover, since relatively few biologists are experienced with SQL or programming, both Ensembl and UCSC also provide Web-based user interfaces for batch-querying by the nonprogrammer. These interfaces, which are suitable for simple queries involving one or two database tables, are referred to as the “Table Browser” at UCSC [11] and “BioMart” at Ensembl [12]. A screen shot of the Table Browser Interface is shown in Figure 1. Using the Table Browser, one can query any table in the UCSC Browser database, restrict the retrieved records to ones satisfying various constraints, perform intersections between tables, and obtain the output data in multiple useful formats. BioMart's capabilities are similar.

So, for example, with the Table Browser, we can answer our earlier question of identifying which of 100 polymorphisms were previously known (as evidenced by dbSNP annotations), in the following manner. First, we would make a custom table (actually called a “custom track” in UCSC database parlance) of the genomic coordinates of our polymorphisms, if necessary obtaining the coordinates from the gene sequences themselves using the UCSC BLAT tool [13]. Then we would intersect our custom table with the appropriate UCSC SNP table (e.g., table “snp126” for the March 2006 build of the UCSC database). The result of the table intersection would be a list of those polymorphisms present in dbSNP.

However, our example also indicates some of the limitations of the Table Browser approach. One problem becomes apparent if we attempt to identify those polymorphisms that are in dbSNP, are also in CpG islands, and also have polymorphism sites that align with ESTs found in other mammals. To identify these polymorphisms, we would need to perform multiple table intersections with the Table Browser. However, performing multiple table intersections within the Table Browser is awkward. To address this problem, we could access the “Galaxy” Web site User Interface [14] (http://g2.bx.psu.edu). Galaxy is a relatively new Web site that provides a set of built-in post-processing tools (including tools from EMBOSS [15], PHYLIP [16], PAML [17], and R [18]) that can act directly on data acquired from the Table Browser, Biomart, and other sources. In particular, the Galaxy User Interface includes a “history” mechanism, which facilitates performing intersections of multiple tables from the Table Browser.

Although Galaxy alleviates the difficulties of multiple table intersections, our example also highlights a more fundamental limitation to the Table Browser approach. This limitation is that answering biological questions often requires data post-processing, and this post-processing may sometimes be more involved than what one can be accomplished using just the Galaxy toolset. For example, to determine if our polymorphisms have been observed in an EST not only requires intersecting our custom table with the EST table, but also obtaining the actual EST sequences, and, finally, inspecting those ESTs at the polymorphism locations. If we have 100 polymorphisms and multiple ESTs overlapping any single one, manually screening all of the ESTs is not feasible. Similarly, the example of searching for candidate ADAR sites by comparing genomic DNA with EST data also requires writing sequence-comparison software in addition to batch-downloading of data.

## Programmatic Batch-Querying and Post-Processing

For more complex types of genomic data analysis, it may be necessary to write one's own computer program or script to perform the required post-processing of the data. Now, in such cases, it is of course possible to simply take the data downloaded from the Table Browser or Biomart and analyze it with software written “from scratch” in any computer language of one's choice. However, this is typically an inefficient approach. Rather, it is generally preferable to use the extensive API libraries of data extraction and manipulation tools that have already been developed by the Browser Teams for these purposes. In this way one has immediate access to functions that perform essentially all the data manipulations that we are accustomed to performing interactively on the respective browsers.

For the UCSC Databases, the principal API is in C and contains a comprehensive collection of utility programs and library routines (http://hgdownload.cse.ucsc.edu/admin/jksrc.zip). These programs were originally developed by Jim Kent and are typically referred to as the “kent source tree.” The included utility programs, which can generally be run either in stand-alone mode or be incorporated into one's own code, include programs for sorting, splitting, or merging fasta sequences; record parsing and data conversion using GenBank, fasta, nib, and blast data formats; sequence alignment; motif searching; hidden Markov model development; and much more. Library subroutines are available for everything from managing C data structures such as linked lists, balanced trees, hashes, and directed graphs to developing routines for SQL, HTML, or CGI code. Additional library functions are available for biological sequence and data manipulation tasks such as reverse complementation, codon and amino acid lookup and sequence translation, as well as functions specifically designed for extracting, loading, and manipulating data in the UCSC Genome Browser Databases. The code is open-source and, except for the browser-specific libraries, is completely free to all. The UCSC-Browser–specific code, while free for academic, research, and personal use, does require licensing for commercial use.

The Ensembl code base has comparable capabilities to UCSC's with APIs implemented for either Perl or Java. The Ensembl software is also open-source and is completely free for all use. See http://www.ensembl.org/info/software/core/index.html for links to more information on the Ensembl APIs.

The main cost of using these databases and their associated software (apart from possible commercial licensing fees) is the time required to learn how to use them and to obtain access to a copy of all or part of the database. The steepness of the learning curve is primarily due to the complexity of the data, but is also because these databases were designed more for interactive querying than for programmed use. In particular, programmed access to the UCSC database can be challenging to learn, since the appropriate table and code documentation are sometimes not easy to locate. For UCSC database table descriptions, often a good place to start is by selecting the table of interest in the Table Browser (http://genome.ucsc.edu/cgi-bin/hgTables) and then selecting “Describe table schema” (see also Figure 1). In contrast, documentation for the source code tends to be embedded in the code itself. The relevant documentation can usually be found by applying the Unix “grep” command or some similar text-finding utility to the library subdirectories of the kent source code tree.

The requirement for access to a database copy exists because the genome browsers themselves do not have the capacity to handle programmatic querying. There are three ways to create such database access: using a public mirror database, downloading individual database tables and/or files, and creating one's own private mirror. For occasional programmatic database-querying, probably the easiest approach is to use direct SQL-querying of the public mySQL mirrors set up for this purpose, using host = genome-mysql.cse.ucsc.edu with username = genome for UCSC, or host = ensembldb.ensembl.org with username = anonymous for Ensembl. Since genome-mysql.cse.ucsc.edu is an (almost) exact mirror of the UCSC browser database, data extraction and manipulation code taken from the kent source tree will typically run without any modification (one important exception is code that accesses actual sequence or alignment data; however, it is not difficult to work around this limitation by locally installing just the sequence and alignment data).

An alternative to using a public mirror, when data from only a small number of database tables is required, is to simply download the needed tables, using either the Table Browser, the UCSC http download site (e.g., http://hgdownload.cse.ucsc.edu/goldenPath/hg18/database), or the UCSC DAS [14] interface. Once the data have been downloaded, they can be loaded into and accessed from a local relational database using programs provided in the kent source tree or the generic genomic browser utility, GBrowse [19]. Alternatively, the data can be read directly into the analysis program, again using code from the kent source tree.

Finally, the third option is to install one's own local mirror of all (or, more likely, part) of the genome database. Although performing such an installation requires a substantial amount of local disk space, is not trivial, and, at first, may even appear daunting, the steps involved are generally well-documented (http://genome.ucsc.edu/admin/mirror.html), and installation should be reasonably straightforward for someone with Unix experience. Installation requirements for building an Ensembl mirror are comparable with those for the UCSC system and are documented at http://www.ensembl.org/info/software/website/index.html. Setting up a private mirror does entail considerable initial effort. Moreover, if one's applications need genome annotations incorporating the most recent database updates, using a public mirror database may be advantageous. On the other hand, maintaining one's own private mirror database does provide several significant advantages. In particular, concern about the restrictions and limitations of using a shared resource are no longer relevant, and, once created, one has a permanent, customizable resource that can be used repeatedly for multiple investigations.

## An Example

To illustrate the power of automated genome-database–querying using the UCSC API, let us consider another simple example that is illustrative and is not unlike more complex and realistic ones (see, for example, the analysis of snoRNA introns in reference [20]). Specifically, let us assume that we have a list of coordinates of some genomic feature (e.g., snoRNAs) located within introns (as noted earlier, if necessary, these coordinates can easily be obtained from the sequences of the features by using BLAT). We further assume that we want to know the median length of these embedding introns and how this length compares with the lengths of other introns of the same genes, in order to test the hypothesis that such intronic features occur in introns with lengths that are longer (or shorter) than the median intron length.

Datasets S1, S2, and S3 contain, respectively, the install and usage instructions, Unix tar file, and source code for a simple, short (less than 250-line) C program solution to this example using the UCSC database and the kent source code. The program is actually significantly longer than necessary since it illustrates all three of the database access methods described above. The program works by first reading in a list of genomic regions stored in a “bed” file and then either reading in a list of gene records from a local file, or else setting up an SQL connection to obtain gene table data from a local or public mirror of the UCSC database. The program then reads one genomic location at a time, obtains the gene exon–intron annotation data for each gene that overlaps the location, and selects the longest overlapping gene. Next, for each of the selected gene's introns, the intron's length is computed and stored in one of two lists, depending on whether the current intron overlaps the region in the bed file or not. Finally, the program computes the median length of the introns that overlapped regions in the input list (i.e., that overlap the feature) and the median length of the introns that did not.

**Figure 2:**
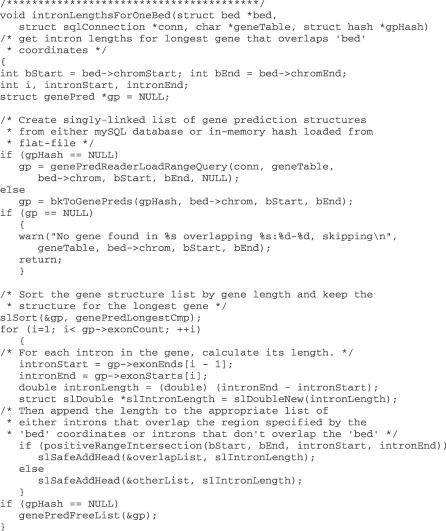
Subroutine Calculating Intronic Lengths, Illustrating C Structures, and Library Routines from the Kent Source Code Tree Subroutine is part of the sample program in Dataset S3.

We now look at the code for the principal subroutine of the program (shown in Figure 2) in a bit more detail. The routine uses several standard kent C code structures. These include structures for an SQL connection (conn), for a singly linked list of “bed” locations (bed), for a list of gene structures (gp), for a hash (gpHash), and, implicitly as global variables, for lists of floating point numbers (overlapList and otherList). Definitions of these structures can be found in the kent source code “include” files jksql.h, bed.h, genePred.h, hash.h, and common.h, respectively.

With these definitions, the subroutine first creates a list of all gene structures for all genes that overlap the input bed region. If the gene-data access method is via a mirror database (indicated by gpHash being null), obtaining this list of gene structures requires only a call to the library routine genePredReaderLoadRangeQuery (defined in genePredReader.h). If, instead, gene-table access is via a local file, one needs to write a short additional access routine bkToGenePreds (shown in Dataset S3), which is based on a “binKeeper-hash” data structure. This data structure has two levels. The higher level is a simple hash in which the keys are chromosome names and the values are pointers to binKeeper structures (defined in binKeeper.h). The binKeeper structures are used to store chromosome feature data (in this case gene structures) in “bins,” such that a feature spanning a coordinate range is stored in the smallest bin that completely contains the specified region. This type of implementation enables very fast data retrieval.

Once the list of overlapping genes is retrieved, the longest one is found by simply calling the linked-list sort function, slSort. (One does need to write a short comparison function, here called genePredLongestCmp, specifying that the gene sorting is based on transcript length.) Next, the program is ready to cycle through all of the exons of the longest overlapping gene and to compute intron lengths by simple subtraction. Then the code determines whether the intron overlaps the input bed using the function positiveRangeIntersection, and, depending on the result of the intersection test, appends the length to either of the linked lists, overlapList or otherList, using the library routine slSafeAddHead. Finally, the memory allocated for the gene structures is freed.

By examining this code, it should be apparent that, once the initial learning curve has been overcome, using the UCSC database and the kent source code can turn what would otherwise be a rather large programming project into a relatively straightforward exercise.

## Summary

Web-based, interactive querying of the genome databases has enabled the analysis of genomes in an integrated and visual manner that previously was difficult or impossible. However, many important biological questions cannot practically be answered using simple interactive methods that query only a single genomic location at a time. Addressing these questions requires batch- and programmatic database-querying. Although these approaches involve an initial, one-time cost of learning how to use the associated API and establishing an access method for programmed querying, the important capabilities they provide for addressing significant and otherwise relatively complex genomic questions often makes this effort well worthwhile. Moreover, as Web-based interfaces like Galaxy—which provide batch data acquisition and post-processing, but do not require programming—evolve, the powerful tools of genome-wide data analysis should become accessible to an ever wider range of biologists.

## Supporting Information

Dataset S1 Software README File Installation and usage instructions for the included sample software.
(5 KB TXT)

Dataset S2 gbdExample-0.1.tar.gz—Software Source Code Tar File Code for the example described in the article text.
(445 KB TAR)

Dataset S3 gbdExample.c—Source Code of the Example Software Described in the Text (7 KB TXT)

## Abbreviations:

ADAR: adenosine deaminase enzymes that convert specific adenosines in RNA to inosine
API: application programmer interface
dbSNP: Single Nucleotide Polymorphism Database
EST: expressed sequence tag
MGD: Mouse Genome Database
NCBI: National Center for Biotechnology Information
SGD: Saccharomyces Genome Database
SQL: Structured Query Language
UCSC: University of California Santa Cruz
